# Feasibility and physiological effects of a combined exercise and nutritional intervention in older adults with cancer under catabolic stress

**DOI:** 10.3389/fphys.2026.1779559

**Published:** 2026-04-01

**Authors:** Song Ee Park, Jin Hwa Choi, Du Hwan Kim, Don-Kyu Kim, Yongsoon Park, Yong Chan Ha, In Gyu Hwang

**Affiliations:** 1 Division of Medical Oncology, Department of Internal Medicine, Seoul St. Mary’s Hospital, College of Medicine, The Catholic University of Korea, Seoul, Republic of Korea; 2 Department of Radiation Oncology, Chung-Ang University College of Medicine, Seoul, Republic of Korea; 3 Chung-Ang University Integrated Oncology and Palliative Care Research Institute, Seoul, Republic of Korea; 4 Department of Rehabilitation Medicine, Chung-Ang University College of Medicine, Seoul, Republic of Korea; 5 Department of Food and Nutrition, Hanyang University, Seoul, Republic of Korea; 6 Department of Orthopedic Surgery, Seoul Bumin Hospital, Seoul, Republic of Korea; 7 Department of Internal Medicine, Chung-Ang University College of Medicine, Seoul, Republic of Korea

**Keywords:** ageing, body composition, exercise physiology, nutritional intervention, sarcopenia, skeletal muscle

## Abstract

**Background:**

Ageing and cancer are associated with accelerated skeletal muscle catabolism, leading to sarcopenia, adverse body composition changes, and functional decline. Exercise and nutritional support are established physiological countermeasures against muscle loss; however, their feasibility and physiological effects in older adults exposed to profound catabolic stress remain insufficiently characterized.

**Methods:**

Between 2021 and 2023, adults aged ≥65 years with advanced cancer undergoing systemic therapy participated in a 12-week combined exercise and nutritional intervention program. Participants were assigned to an intervention group (exercise plus nutrition, n=20) or a usual-care control group (n=40). The primary endpoint was feasibility, defined as ≥50% adherence to both exercise and nutritional components during the first 6 weeks. Secondary endpoints included changes in skeletal muscle index (SMI), subcutaneous and visceral fat compartments, safety, and patient-reported quality of life.

**Results:**

The median age was 72 years, 65% were men, and 41.7% had baseline sarcopenia. Adherence rates were 65% for exercise and 75% for nutritional support, with an overall attrition rate of 5% and no intervention-related adverse events. At 6 weeks, SMI declined significantly in the control group, whereas no statistically significant change in SMI was observed in the intervention group. Subcutaneous fat decreased significantly in the intervention group, with a reduction in visceral fat observed among men. At 12 weeks, body composition parameters remained relatively stable in both groups. Modest improvements were noted in fatigue, appetite loss, and nausea.

**Conclusions:**

A combined exercise and nutritional intervention was feasible, safe, and well tolerated in older adults exposed to severe catabolic stress. The preservation of skeletal muscle mass and favorable body composition changes observed suggest meaningful physiological adaptation, supporting exercise and nutritional support as viable physiological countermeasures in vulnerable ageing populations.

## Introduction

Sarcopenia is defined as the progressive and generalized loss of skeletal muscle mass and function and is highly prevalent among older adults with cancer. This condition is particularly common in gastrointestinal malignancies, with reported prevalence rates of 47–60% prior to treatment initiation (Zhang et al., 2016; Lee et al., 2018; Park et al., 2018a, 2020). Age-related declines in muscle mass and strength are further exacerbated by cancer-related metabolic alterations, rendering older adults especially susceptible to sarcopenia. Clinically, sarcopenia is associated with adverse outcomes, including chemotherapy intolerance, dose reductions, treatment delays, and reduced overall survival (Prado et al., 2008; Martin et al., 2013; Caillet et al., 2017). Importantly, cancer-associated muscle wasting cannot be fully reversed by conventional nutritional support alone, underscoring the need for early, multimodal physiological interventions (Kumar et al., 2010).

Older adults with cancer are exposed to a convergence of ageing-related anabolic resistance, systemic inflammation, and treatment-induced catabolic stress. Cytotoxic chemotherapy further amplifies these processes, accelerating skeletal muscle degradation, impairing physical performance, and reducing physiological resilience (Mizrahi et al., 2015; Ligibel et al., 2016). Despite this vulnerability, interventional evidence targeting sarcopenia in older oncology populations remains limited. A recent meta-analysis demonstrated that fewer than 25% of participants enrolled in oncology exercise trials were aged 65 years or older (Dale et al., 2023), highlighting a substantial evidence gap in a population at highest risk of muscle loss.

Exercise therapy represents a cornerstone physiological countermeasure against sarcopenia. Supervised resistance and aerobic exercise programs have been shown to improve muscle strength, fatigue, and quality of life (QoL) in selected cancer populations (Blauwhoff-Buskermolen et al., 2016). However, data in older adults with advanced cancer are scarce, as fatigue, anorexia, treatment-related toxicity, and functional decline often limit adherence to structured exercise regimens. Studies conducted in metastatic breast cancer and recurrent ovarian cancer have reported heterogeneous outcomes, reflecting differences in patient selection, intervention intensity, and concurrent systemic therapy (Stene et al., 2015; Solheim et al., 2017). Furthermore, relatively few trials have implemented combined exercise and nutritional strategies, despite emerging evidence suggesting additive or synergistic effects on muscle preservation and metabolic regulation (McKendry et al., 2020).

We previously conducted a feasibility study (KCT0005615) demonstrating that a 6-week home-based exercise program was safe and achievable in patients with advanced gastrointestinal cancer receiving palliative chemotherapy. However, this earlier study primarily enrolled younger patients (median age, 60 years) and did not incorporate structured protein supplementation, a critical component of sarcopenia mitigation in older adults due to age-related anabolic resistance.

The present prospective study was designed to address these limitations by evaluating the feasibility and preliminary physiological effects of a 12-week structured intervention combining individualized resistance and aerobic exercise with evidence-based protein supplementation in adults aged 65 years and older with advanced cancer. The protein intake protocol was developed in accordance with current guidelines and Korean population data to ensure adequacy in this age group (Park et al., 2018b). The primary objective was to assess program feasibility in terms of adherence and attrition, while secondary endpoints included changes in skeletal muscle index (SMI), body composition, physical function, and QoL.

## Methods

### Patients

Patients aged 65 years or older with histologically confirmed advanced cancer who were scheduled to undergo palliative chemotherapy were eligible for inclusion. Additional eligibility criteria included an Eastern Cooperative Oncology Group (ECOG) performance status of 0–2, expected survival of at least three months, and the ability to provide written informed consent. Patients with cognitive impairment or physical disabilities requiring movement restrictions were excluded. This study was not a randomized trial. Patients who declined to participate in the intervention were enrolled in the control group. For each patient in the intervention group, a comparable control patient was selected based on age, sex, and cancer type. The study was registered with the Clinical Research Information Service (CRIS; KCT0006588) and approved by the Institutional Review Board of Chung-Ang University Hospital (IRB No. 2130-007-455).

### Recruitment and group allocation

Patients were consecutively recruited from older adults receiving palliative chemotherapy at Chung-Ang University Hospital between 2021 and 2023. Participation in the exercise and nutritional intervention was voluntary. Patients who declined participation due to personal preference, logistical constraints, or physical limitations were assigned to the control group. As a result, the unequal group sizes reflect the pragmatic, real-world nature of this study rather than a predefined allocation ratio. To minimize selection bias, control patients were selected to be comparable to intervention patients with respect to age, sex, and cancer type.

### Exercise intervention

The participants in the intervention group underwent a 12-week structured exercise program in conjunction with palliative chemotherapy. The exercise program was developed in collaboration with the Department of Rehabilitation Medicine. Before chemotherapy initiation, eligible patients underwent baseline physical assessments and personalized education regarding exercise protocols. The program comprised three progressive intensity levels: low, moderate, and high, allowing participants to start comfortably and increase the intensity as tolerated. Participants were encouraged to begin at the low-intensity level and to progress to a higher level when tolerated. Each session included a 5-minute warm-up, 20–30 minutes of aerobic and resistance exercises (e.g., treadmill walking, stationary cycling, dumbbell, or resistance band training), and a 5-minute cool-down, totaling 30–40 minutes per session. At the low-intensity level, the total session duration was 30 minutes, including 20 minutes of main exercise, whereas the moderate- and high-intensity levels were performed for 40 minutes, including 30 minutes of main exercise. Resistance exercises targeted major muscle groups and consisted of six upper-body exercises (front raises, lateral raises, lateral pull-down, biceps curl, triceps extension, and upright row) and six lower-body exercises (squats, leg press, leg abduction, knee flexion, knee extension, and calf raising). The main exercise component combined aerobic and resistance training; aerobic exercise included treadmill walking, level walking, or running, and resistance exercise was performed using dumbbells, isotonic resistance bands, or a bicycle ergometer. Exercise intensity was guided by the Rating of Perceived Exertion (RPE) and Heart Rate Reserve (HRR) as follows: low (RPE 9–10, HRR ~30%), moderate (RPE 11–13, HRR 40–50%), and high (RPE 14–15, HRR ~70. The structure and implementation of the program were based on previously validated exercise protocols (Park et al., 2023). All participants received exercise education from rehabilitation specialists during chemotherapy cycle 1. Supervised sessions were scheduled only on days 1–3 of cycle 1 as an instructional and reinforcement period.

### Nutritional intervention

Nutritional counseling was performed by clinical dietitians who assessed baseline protein intake through a 3-day 24-hour dietary recall. The target protein intake was set to 1.2 g/kg/day. Dietary protein deficiencies were identified, and whey protein supplementation (Korea Medical Foods; 9 g protein per 10 g powder, 404 kcal) was prescribed to meet the individual protein targets. Supplementation was integrated into daily meals, including beverages or soft foods. Nutritional intervention followed a previously published protocol (Park et al., 2018b).

### Endpoints

The primary endpoints were intervention compliance and attrition rates. Exercise compliance was stratified into three categories: <50%, 50–80%, and >80%, with ≥50% defined as acceptable. Completing 30 sessions over six weeks (five sessions per week) resulted in 100% compliance. Nutritional compliance was defined as the percentage of protein supplement intake calculated at the end of the 6-week intervention period using the following formula: (total number of sachets provided – number of sachets returned)/total number of sachets provided × 100. As with exercise compliance, nutritional compliance of ≥50% was defined as acceptable. Compliance, adverse effects, and safety were monitored biweekly during the 12-week intervention period.

The secondary endpoints included changes in body composition, including SMI, bioelectrical impedance (InBody), body weight, body mass index (BMI), handgrip strength, and six-minute walk test results. QoL was measured using the European Organization for Research and Treatment of Cancer Quality of Life Questionnaire Core 30 at baseline, six weeks, and 12 weeks. Scores were transformed to a 0–100 scale, with higher scores indicating better function or more severe symptoms, depending on the domain.

For body composition analysis, the visceral fat area (VFA), subcutaneous fat area (SCFA), and skeletal muscle area (SMA) were measured using commercial imaging software (TeraRecon Aquarius, TeraRecon, USA). A single axial computed tomography (CT) image at the L3 level with both transverse processes visible was selected for automatic calculation. Based on predefined Hounsfield unit (HU) ranges, the software measured the SMA (-29 to +150 HU), VFA (-150 to -50 HU), and SCFA (-190 to -30 HU) (Aubrey et al., 2014). The SMI was calculated by dividing the SMA at L3 by the square of the patient’s height (cm²/m²) and was used as a validated surrogate for estimating whole-body skeletal muscle mass. Sarcopenia was defined using Korean population-specific cutoffs of SMI <49 cm²/m² for men and <31 cm²/m² for women (Kim et al., 2012).

### Sample size consideration

This study was intentionally designed as a feasibility-oriented and exploratory physiological intervention study to evaluate the safety, tolerability, and adherence of a combined exercise and nutritional program in older adults exposed to significant catabolic stress due to advanced cancer. As the primary objective was to assess feasibility rather than to test a predefined efficacy hypothesis, a formal sample size calculation or power analysis was not performed.

Instead, the target sample size was pragmatically determined based on the number of eligible participants who could be recruited within the predefined study period at a single center. This approach is consistent with methodological recommendations for feasibility and pilot studies, where sample size is guided by the ability to estimate adherence, attrition, and safety outcomes, and to generate preliminary physiological effect estimates rather than to achieve statistical power.

The resulting sample size was considered sufficient to evaluate feasibility-related endpoints and to provide effect size estimates for skeletal muscle and body composition outcomes, thereby informing the design of future adequately powered randomized controlled trials.

### Statistical analysis

Statistical analyses were conducted using SPSS software (version 24.0; IBM Corp., Armonk, NY, USA). Significance was set at P<0.05. Continuous variables are expressed as the means ± standard deviations (SDs) or medians with interquartile ranges (IQRs) based on distribution. Categorical variables are presented as frequencies and percentages. Group comparisons for continuous variables were performed using independent t-tests or Mann–Whitney U tests, whereas categorical variables were assessed using chi-square or Fisher’s exact tests.

As appropriate, body composition and function changes were analyzed using paired t tests or repeated-measures ANOVA. Between-group differences in mean changes were evaluated using analysis of covariance after adjusting for baseline values. Longitudinal changes in the SMI and other variables were analyzed using linear mixed-effects models. Given the exploratory nature of this study, all statistical analyses were considered descriptive and hypothesis-generating, and results should be interpreted accordingly.

## Results

### Patients

Sixty patients were enrolled and assigned to either the intervention group (n=20) or the control group (n=40). As shown in **Table 1**, the median age was significantly lower in the intervention group than in the control group (68 vs. 72 years, P = 0.013). The proportion of male patients did not significantly differ between the groups (75.0% vs. 60.0%, P = 0.251). The proportion of patients with an ECOG performance status of 0–1 was significantly higher in the intervention group than in the control group (95.0% vs. 70.0%, P = 0.027). The BMI, SMI, at the L3 level, and prevalence of sarcopenia were comparable between the groups. The distribution of palliative chemotherapy lines differed significantly (P = 0.039), with more patients in the intervention group receiving secondary or later treatments. Other baseline characteristics, including cancer type, were well balanced between the two groups.

**Table 1:** Baseline characteristics.

Characteristics	Total	Exercise and nutrition group	Control	p value
	(N = 60)	(n=20)	(n=40)	
Age (years)				
Median	72	68	72	0.013
Range	65–88	65–82	65–88	
Sex, n (%)				
Male	39 (65.0%)	15 (75.0%)	24 (60.0%)	0.251
ECOG PS, n (%)				
0–1	47 (78.3%)	19 (95.0%)	28 (70.0%)	0.027
2	13 (21.7%)	1 (5.0%)	12 (30.0%)	
Height (cm), mean ± SD	161.2 ± 7.9	162.8 ± 5.8	160.4 ± 8.8	0.292
Weight (kg), mean ± SD	57.8 ± 11.0	60.2 ± 9.5	56.6 ± 11.7	0.244
BMI, kg/m^2^, mean ± SD	22.1 ± 3.6	22.7 ± 3.7	21.8 ± 3.6	0.374
Underweight <20	18 (30.0%)	4 (20.0%)	14 (35.0%)	0.449
Normal 20–24.9	30 (50.0%)	12 (60.0%)	18 (45.0%)	
Overweight ≥25	12 (20.0%)	4 (20.0%)	8 (20.0%)	
L3 Skeletal muscle index (cm^2^/m^2^)	43.0 ± 7.4	44.8 ± 9.0	42.1 ± 6.4	0.187
Sarcopenia, n (%)	15 (41.7%)	8 (40.0%)	7 (43.8%)	0.821
Primary cancer				0.573
Stomach cancer	12 (20.0%)	5 (25.0%)	7 (17.5%)	
Esophageal cancer	2 (3.3%)	1 (5.0%)	1 (2.5%)	
Colorectal cancer	21 (35.0%)	6 (30.0%)	15 (37.5%)	
Biliary pancreas cancer	18 (30.0%)	7 (35.0%)	11 (27.5%)	
Lung cancer	6 (8.4%)	0 (0%)	5 (12.5%)	
Other cancer	2 (3.3%)	1 (5.0%)	1 (2.5%)	
Palliative chemotherapy line				0.039
1st line	40 (66.7%)	13 (65.0%)	27 (67.5%)	
2nd line	13 (21.7%)	2 (10.0%)	11 (27.5%)	
≥3rd line	7 (11.6%)	65(25.0%)	2 (5.0%)	

ECOG PS, Eastern Cooperative Oncology Group performance status; SD, standard deviation; BMI, body mass index.

### Compliance

**Figure 1:**
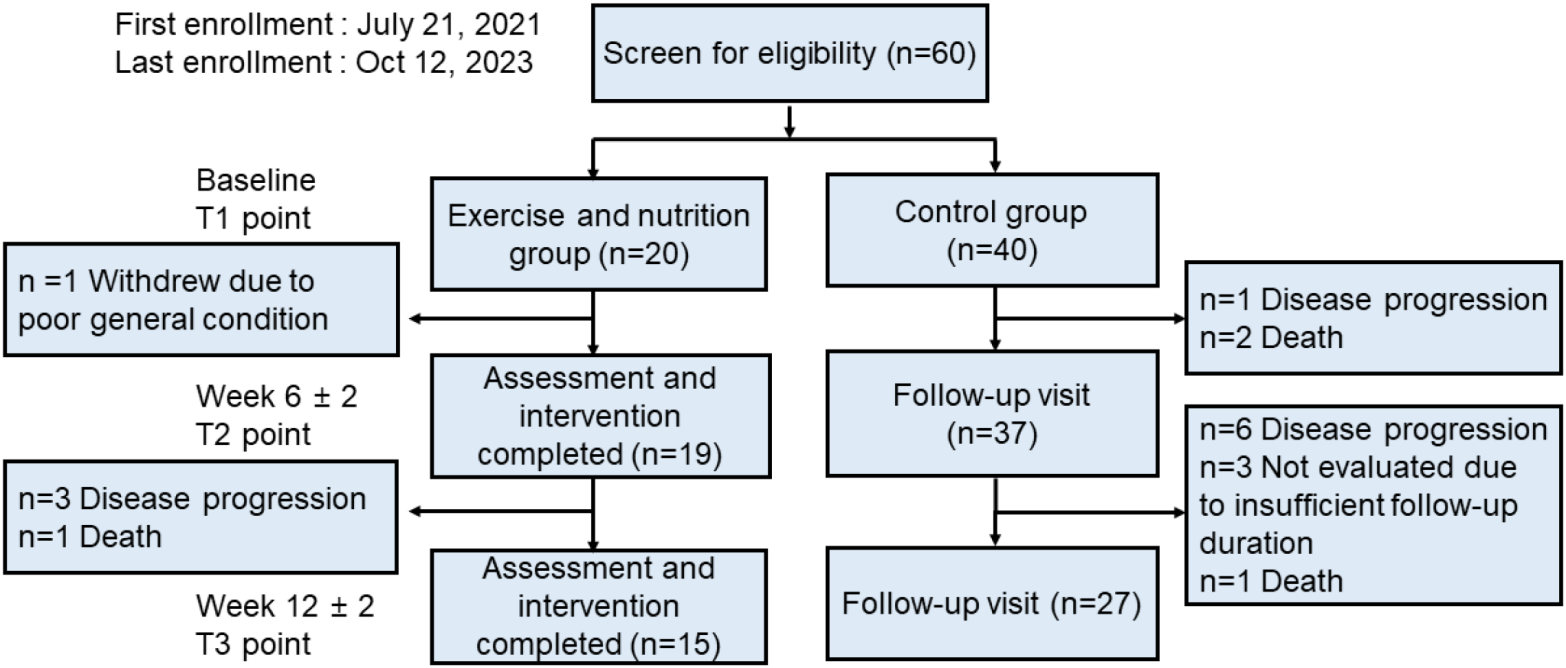
Patient flowchart.

Twenty patients were analyzed at the start of the study (**Figure 1**). In the intervention group, one patient withdrew because of a poor general condition. The attrition rate was 5.0% (1/20). The exercise compliance rate was 65% (13/20) during the six weeks (**Table 2**). During chemotherapy cycle 1, 27 of 60 prescribed supervised sessions scheduled on days 1–3 (45.0%) were completed. During the subsequent home-based period, corresponding to days 4–14 of the exercise schedule, 132 of 220 prescribed sessions (60.0%) were completed. Over six weeks, the median number of exercise sessions was 28 (range 0–42). The mean time per session was 50.3 ± 8.4 minutes. Seven (35.0%) patients completed more than 80% of the resistance and aerobic exercises. Seventy-five percent of patients maintained the intervention for 12 weeks (15/20). The nutritional compliance rate was 75.0% (15/20). Eight patients (40.0%) achieved >80% adherence to the protein supplementation. Based on individualized dietary assessments, two patients achieved sufficient protein intake through meals and did not require supplementation (0 packs/day) (**Supplementary Table 3**).

**Table 2:** Primary endpoint: adherence to the exercise and nutritional intervention.

Adherence to the individual intervention component	<50%		50–79%		≥80%	
	n	%	n	%	n	%
Oral protein supplement (n=20)	5	25	7	35	8	40
Resistance (n=20)	13	65	4	20	3	15
Aerobic (n=20)	7	35	7	35	6	30
Adherence to combined intervention components	<50%		50–79%		≥80%	
Aerobic + Resistance	13	65	5	25	2	10
Aerobic + Protein	9	45	7	35	4	20
Resistance + Protein	14	70	5	25	1	5
Aerobic + Resistance + Protein	14	70	5	25	1	5

### Exercise adherence during chemotherapy cycles

**Figure 2:**
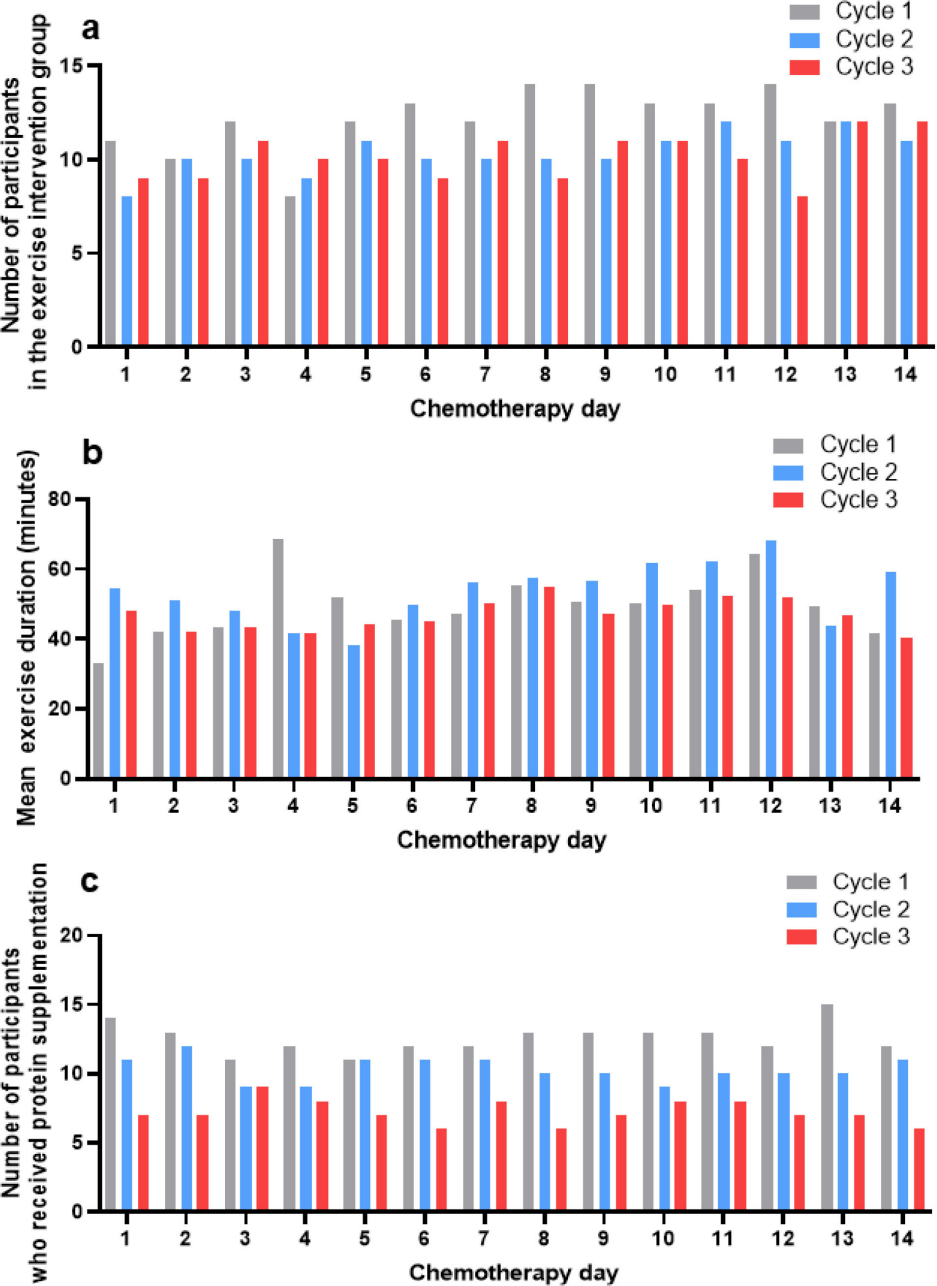
Exercise characteristics in the intervention group. **(a)** The number of patients who exercised and the number of chemotherapy days according to the cycle, **(b)** the mean exercise time and the number of chemotherapy days according to the cycle, and **(c)** the number of patients taking protein supplements for each chemotherapy cycle.

The median number of patients who participated in exercise sessions during the first and second chemotherapy cycles was comparable at 12 (range, 8–14) and 10 (range, 8–12) patients per day, respectively. However, exercise participation declined significantly in cycles 2 and 3 compared with cycle 1 (cycle 1 vs 2, P = 0.002; cycle 1 vs 3, P = 0.001). Participation remained similar between cycle 2 and 3, with a median of 10 (range, 8–12) patients per day (**Figure 2a**). The mean exercise time was 50.4 minutes in the first cycle, 53.3 minutes in the second cycle, and 47.2 minutes in the third cycle. Exercise time significantly decreased in the third cycle compared to that in the first and second cycles (P = 0.010) (**Figure 2b**). In all cycles, patients participated in more aerobic exercise than in resistance exercise, and a progressive decline in total exercise adherence was observed as chemotherapy progressed (**Supplementary Figure 2**). During the first, second, and third cycles, 65.3%, 51.8%, and 63.7% of patients performed moderate-intensity exercises, respectively (**Supplementary Figures 4a–c**). During the first three days of chemotherapy, most patients performed exercises at the hospital. After day four of chemotherapy, 19.0% of the patients performed the exercises at home or near home (**Supplementary Figures 4d–f**). Among the patients, 17.9% exercised at a playground or park during cycle 1, while 17.1% participated in other (hiking group) (**Supplementary Figures 4d–f**).

### Nutrition adherence during chemotherapy cycles

The median numbers of patients who received protein supplementation were 12, 10, and 7 in the first, second, and third cycles, respectively (**Figure 2c**). Although the number of patients gradually decreased over successive cycles, adherence was generally consistent. However, there were no significant differences in compliance rates across the three cycles (P = 0.167).

### Body composition and physical function

At six weeks, the control group experienced a significant decrease in SMI (-1.3 ± 3.0, P = 0.010), whereas SMI was maintained in the intervention group (-3.3 ± 12.0, P = 0.234) (**Table 3**). SCFA significantly decreased in the intervention group (-11.0 ± 20.4, P = 0.030), especially among women (-25.4 ± 22.7, P = 0.067). VFA reduction trends were noted (-10.5 ± 26.9, P = 0.105), with significant reductions in men (-17.5 ± 26.8, P = 0.031). At 12 weeks, body composition parameters remained relatively stable in both groups, with no statistically significant changes in SMI or adipose tissue levels.

**Table 3:** Changes in body composition at 6 and 12 weeks between the intervention and control groups.

	Baseline		Change from baseline (6 weeks)				Change from baseline (12 weeks)			
	Intervention group (n=20)	Control group (n=40)	Intervention group (n=19)	p value	Control group (n=37)	p value	Intervention group (n=15)	p value	Control group (n=27)	p value
	Mean	Mean	Mean ± SD		Mean ± SD		Mean ± SD		Mean ± SD	
SMI (cm^2^/m^2)^	44.8	42.1	-3.3 ± 12.0	0.234	-1.3 ± 3.0	0.010	- 0.6 ± 2.8	0.413	-0.4 ± 3.7	0.546
Male	46.9	44.6	-4.4 ± 13.9	0.259	-1.2 ± 2.9	0.062	-0.4 ± 3.0	0.604	-0.7 ± 2.9	0.303
Female	38.5	38.3	-0.5 ± 1.6	0.493	-1.5 ± 3.3	0.093	-1.1 ± 2.1	0.430	0.1 ± 5.2	0.941
Subcutaneous fat (cm^2^)	108.1	98.7	-11.0 ± 20.4	0.030	-4.1 ± 22.2	0.268	-6.2 ± 38.5	0.538	2.4 ± 26.1	0.633
Male	78.9	100.1	-5.9 ±17.7	0.232	-1.3 ± 17.9	0.737	5.4 ± 33.8	0.591	6.3 ± 19.5	0.183
Female	195.8	96.6	-25.4 ± 22.7	0.067	-8.2 ± 27.5	0.267	-53.0 ± 2.6	0.001	-5.4 ± 36.1	0.661
Visceral fat (cm^2^)	94.1	131.5	-10.5 ± 26.9	0.105	-9.9 ± 31.4	0.061	-18.1 ± 33.8	0.057	-2.0 ± 30.6	0.736
Male	95.6	155.7	-17.5 ± 26.8	0.031	-10.3 ± 36.5	0.198	-18.8 ± 37.6	0.110	-2.0 ± 30.5	0.781
Female	89.4	95.1	8.4 ± 18.0	0.358	-9.2 ± 22.0	0.127	-15.0 ± 14.1	0.208	-1.9 ± 32.7	0.861

SMI, skeletal muscle index.

Body composition parameters measured using InBody, including skeletal muscle mass, fat mass, and lean body mass, did not change significantly over the 12-week period (**Table 4**). Similarly, hand grip strength and 6-meter gait speed remained stable, indicating that overall physical function and muscle mass were maintained during the intervention (**Table 4**).

**Table 4:** Body composition values and physical function changes.

	Baseline (n=20)		6 weeks (n=19)		12 weeks (n=15)		Mean change over 6 weeks			Mean change over 12 weeks		
Measure	Mean	SD	Mean	SD	Mean	SD	Mean	95% CI	p value	Mean	95% CI	p value
Body weight (kg)	60.2	9.5	59.2	10.2	62.0	11.3	−1.31	−2.56 to −0.05	0.055	0.06	-2.26 to 2.38	0.957
BMI (kg/m^2^)	22.7	3.7	22.2	4.2	23.1	4.6	-0.57	-1.04 to -0.1	0.029	-0.07	-0.91 to 0.76	0.868
Skeletal muscle mass (Inbody)	24.0	4.1	23.8	4.1	25.0	4.2	-0.65	-1.51 to 0.22	0.132	0.31	-0.8 to 0.41	0.333
Fat mass, kg (Inbody)	15.0	6.8	14.4	6.3	15.7	8.1	-0.67	-2.1 to 0.75	0.558	-0.68	-2.36 to 0.00	0.398
Lean body mass, total body (Inbody)	44.6	6.6	44.6	6.7	46.2	7.0	-0.86	-2.39 to 0.67	0.254	0.74	-1.22 to 2.71	0.428
Hand grip test	23.1	6.0	22.1	7.0	23.3	6.2	-0.99	-2.79 to 0.82	0.299	0.02	-2.13 to 2.18	0.983
6-meter walk test	6.4	1.6	6.3	1.4	6.1	1.3	-0.17	-0.81 to 0.47	0.613	-0.3	-1.02 to 0.42	0.828

BMI, body mass index; SD, standard deviation; CI, confidence interval.

### Quality of life and adverse events

**Figure 3:**
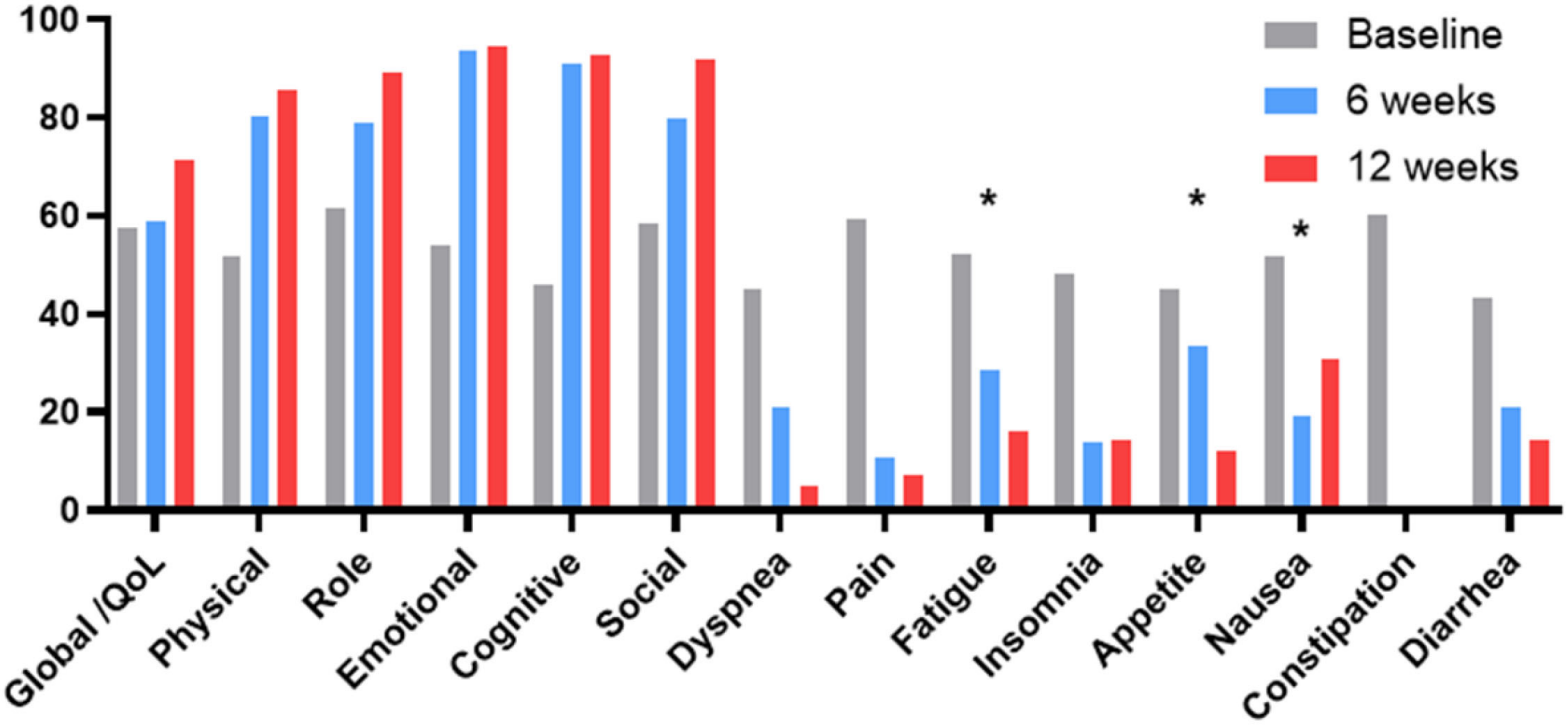
Changes in quality of life (QoL) scores over time following a combined exercise and nutritional intervention in patients with cancer. *indicates a significant improvement compared with baseline (P<0.05).

Quality of life (QoL) was evaluated within the intervention group using the EORTC QLQ-C30 questionnaire at baseline, 6 weeks, and 12 weeks. At 6 weeks, overall QoL showed a slight improvement (57.5 to 58.8, P = 0.652), with a greater improvement at 12 weeks (71.4, P = 0.095). Pain scores decreased markedly over time (59.2 to 10.5 at 6 weeks and 7.1 at 12 weeks), indicating a trend toward significance (P = 0.089 and P = 0.068, respectively). Fatigue significantly improved from 52.2 at baseline to 15.9 at 12 weeks (P = 0.009). Appetite improved significantly by 12 weeks (45.0 to 11.9, P = 0.002), and nausea improved from 51.7 to 30.9 (P = 0.026). Functional domains, including physical, role, emotional, cognitive, and social functioning, improved after 12 weeks, although the differences were not significant. Dyspnea improved early, whereas insomnia and gastrointestinal symptoms remained relatively unchanged (**Figure 3**, **Supplementary Table 1**). No adverse events, including hepatic or renal toxicities related to chemotherapy agents or protein supplementation, were observed during the study period.

## Discussion

This study demonstrates the feasibility of a combined exercise and nutritional intervention in older adults with cancer undergoing systemic therapy and exposed to substantial catabolic stress. Adherence rates of 65.0% for exercise and 80.0% for nutritional supplementation indicate that structured physiological interventions can be implemented and sustained in this vulnerable population. Importantly, the intervention was associated with relatively stable skeletal muscle mass, reductions in subcutaneous fat, and improvements in patient-reported quality of life (QoL), supporting the role of integrative physiological countermeasures in mitigating cancer- and treatment-related muscle deterioration.

Exercise adherence in the intervention group was comparable to that reported in prior studies, with a median of 23 sessions over six weeks and a mean session duration of approximately 50 minutes. These findings are consistent with previous reports demonstrating adherence rates of approximately 63% with similar intervention intensity, despite minor differences in session frequency and duration (Park et al., 2023). Although exercise participation declined during later chemotherapy cycles, participants who continued to increase exercise duration exhibited sustained motivation. Poor adherence was more frequently observed among individuals who did not maintain exercise routines at home, highlighting the importance of structured follow-up and behavioral reinforcement. Simple strategies such as regular monitoring, scheduled check-ins, or the use of digital engagement tools may enhance long-term adherence and intervention sustainability. Future studies should explore additional supportive strategies, such as adaptive exercise scheduling or fatigue-management approaches, to prevent declines in participation during later chemotherapy cycles.

Adherence to protein supplementation remained stable throughout the chemotherapy period and met the predefined feasibility criteria. Individualized supplementation strategies based on baseline dietary intake likely contributed to consistent adherence. Practical approaches, including consumption with flavored beverages or incorporation into yogurt, were effective in overcoming taste-related barriers. These findings underscore the practicality and real-world applicability of tailored nutritional strategies as part of multimodal physiological interventions in older adults.

From a physiological perspective, skeletal muscle index (SMI) declined significantly in the control group at six weeks, whereas no statistically significant change in SMI was observed in the intervention group. In addition, subcutaneous fat area was significantly reduced in the intervention group, with sex-specific patterns observed for visceral fat reduction—more pronounced among men. These findings suggest differential metabolic and adipose tissue responses to combined exercise and nutritional interventions and highlight the potential value of individualized approaches. Overall, the observed changes align with prior evidence indicating that resistance and aerobic exercise, when combined with adequate nutritional support, can attenuate muscle loss during cancer treatment.

In addition, the reduction in subcutaneous adipose tissue observed in the intervention group should be interpreted alongside the relative stability of skeletal muscle mass. In patients with advanced cancer, simultaneous loss of muscle and adipose tissue may indicate cancer-related cachexia; however, in this study, skeletal muscle mass remained relatively stable while subcutaneous adipose tissue decreased. This pattern may reflect favorable body recomposition associated with increased physical activity rather than disease-related wasting. The absence of significant changes in overall body weight likely reflects offsetting changes between adipose tissue and lean mass.

Compared with previous studies predominantly enrolling younger patients, this study included a broader older population and observed slightly lower adherence rates, largely attributable to treatment-related fatigue and comorbid conditions. While some older adults demonstrated exercise adherence and performance comparable to younger cohorts, resistance exercise adherence was lower than that reported in prior studies involving nonelderly patients with gastrointestinal cancer. This discrepancy emphasizes the need for age-adapted resistance training protocols that account for physical limitations, treatment burden, and recovery capacity. Lower-impact resistance modalities, combined with individualized progression and structured support, may improve feasibility and long-term adherence in older adults.

Performance status also influenced intervention feasibility. In this cohort, patients with an Eastern Cooperative Oncology Group (ECOG) performance status of 0–1 were generally able to adhere to the intervention when adequate support was provided, whereas one patient with an ECOG score of 2 discontinued participation due to functional limitations. These findings suggest that while structured exercise and nutritional interventions are feasible for patients with preserved functional status, those with greater impairment may require more flexible, closely supervised, and individualized approaches.

Measures of physical function, including hand grip strength and gait speed, remained stable throughout the intervention period, indicating overall stability of functional capacity during systemic therapy. Additionally, QoL improved meaningfully by 12 weeks, with significant reductions in fatigue, appetite loss, and nausea, as well as sustained improvements in pain and overall QoL scores. These findings highlight the clinical relevance of symptom relief and functional well-being, even in the absence of survival benefits, and reinforce the value of physiological interventions aimed at improving lived experience in older adults with cancer.

Several limitations should be acknowledged. The heterogeneity of cancer types and the modest sample size limited statistical power for subgroup analyses. In addition, the relatively small sample size may have contributed to baseline imbalances between groups, particularly in age and ECOG performance status. Although statistical adjustments were performed, the younger age and better functional status observed in the intervention group may have provided greater physiological resilience to catabolic stress, which should be considered when interpreting the results. Nevertheless, the consistency of observed trends supports the biological plausibility and feasibility of this approach. Larger, adequately powered trials with longer follow-up are warranted to confirm these findings, assess long-term physiological outcomes, and identify patient subgroups most likely to benefit.

In conclusion, this study demonstrates that a combined exercise and nutritional intervention is feasible and well tolerated in older adults with cancer receiving systemic therapy. The intervention was associated with relatively stable skeletal muscle mass, favorable changes in body composition, and improvements in QoL. These findings support further investigation of lifestyle-based physiological interventions as adjunctive strategies to standard oncological care in ageing populations.

## Data Availability

The raw data supporting the conclusions of this article will be made available by the authors, without undue reservation.

## Supplementary material

Supplementary Table 1 Changes in the European Organization for Research and Treatment of Cancer Quality of Life Questionnaire Core 30 (EORTC QLQ-C30) scores from baseline to week 12 during the exercise and nutrition intervention.

Supplementary Figure 2 The number of patients who exercised according to the type of exercise and chemotherapy day during the six-week intervention.

Supplementary Table 3 Nutrition Compliance Based on Sachet Consumption During the 6-Week Intervention.

Supplementary Figure 4 **(a–c)** Proportion of patients performing low-, moderate-, and high-intensity exercise during each chemotherapy cycle. **(d–f)** Location of exercise (hospital, home/near home, park, other) by chemotherapy day and cycle.
