# Supplementary material for: Feasibility and physiological effects of a combined exercise and nutritional intervention in older adults with cancer under catabolic stress
Source: Front Physiol. 2026 Apr 1;17:1779559. doi: 10.3389/fphys.2026.1779559 (PMC13078977; doi:10.3389/fphys.2026.1779559)

**Supplement**

**Table S1**Changes in the European Organization for Research and Treatment of Cancer Quality of Life Questionnaire Core 30 (EORTC QLQ-C30) scores from baseline to week 12 during the exercise and nutrition intervention

|  | **Baseline (n=20)** | | **6 weeks (n=19)** | | **12 weeks (n=14)** | | **P value  6 weeks** | **P value 12weeks** |
| --- | --- | --- | --- | --- | --- | --- | --- | --- |
|  |  |  |  |  |  |  |  |  |
|  | **Mean** | **SD** | **Mean** | **SD** | **Mean** | **SD** |  |  |
| Global /QoL | 57.5 | 28.9 | 58.8 | 22.8 | 71.4 | 19.3 | 0.652 | 0.095 |
| **Functional** | |  |  |  |  |  |  |  |
| Physical | 51.7 | 13.3 | 80.4 | 16.6 | 85.7 | 12.2 | 0.833 | 0.537 |
| Role | 61.7 | 32.0 | 79.0 | 29.8 | 89.3 | 14.0 | 0.760 | 0.325 |
| Emotional | 54.2 | 16.6 | 93.9 | 8.7 | 94.6 | 9.6 | 0.175 | 0.137 |
| Cognitive | 45.8 | 25.3 | 91.2 | 10.2 | 92.9 | 10.8 | 0.215 | 0.775 |
| Social | 58.3 | 29.9 | 79.8 | 24.6 | 91.7 | 15.7 | 0.261 | 0.486 |
| **Symptom** |  |  |  |  |  |  |  |  |
| Dyspnea | 45.0 | 40.9 | 21.1 | 35.5 | 4.8 | 12.1 | 0.483 | 0.189 |
| Pain | 59.2 | 22.6 | 10.5 | 17.8 | 7.1 | 10.8 | 0.089 | 0.068 |
| Fatigue | 52.2 | 22.3 | 28.7 | 19.4 | 15.9 | 11.3 | 0.757 | 0.009 |
| Insomnia | 48.3 | 35.0 | 14.0 | 20.2 | 14.3 | 25.2 | 0.269 | 0.336 |
| Appetite loss | 45.0 | 46.2 | 33.3 | 38.5 | 11.9 | 21.1 | 0.513 | 0.002 |
| Nausea and vomiting | 51.7 | 26.4 | 19.3 | 27.9 | 31.0 | 24.3 | 0.187 | 0.026 |
| Constipation | 60.0 | 39.9 | 0.0 | 0.0 | 0.0 | 0.0 | N/A | N/A |
| Diarrhea | 43.3 | 34.4 | 21.1 | 29.8 | 14.3 | 28.4 | 1.000 | 0.373 |

**Table S3 Nutrition Compliance Based on Sachet Consumption During the 6-Week Intervention**

| ID | Total sachets provided | Sachets returned | Nutrition compliance |
| --- | --- | --- | --- |
| 1 | 74 | 0 | 100% |
| 2 | 223 | 89 | 60% |
| 3 | 143 | 5 | 97% |
| 4 | 45 | 3 | 93% |
| 5 | 63 | 63 | 0% |
| 6 | 84 | 54 | 36% |
| 7 | 105 | 38 | 64% |
| 8 | 84 | 84 | 0% |
| 9 | 126 | 0 | 100% |
| 10 | 160 | 70 | 56% |
| 11 | 84 | 12 | 86% |
| 12 | 86 | 23 | 73% |
| 13 | 84 | 84 | 0% |
| 14 | 168 | 58 | 65% |
| 15 | 0 | 0 | 100% |
| 16 | 63 | 39 | 38% |
| 17 | 84 | 29 | 65% |
| 18 | 56 | 18 | 68% |
| 19 | 51 | 3 | 94% |
| 20 | 0 | 0 | 100% |

**Figure S2.** The number of patients who exercised according to the type of exercise and chemotherapy day during the six-week intervention


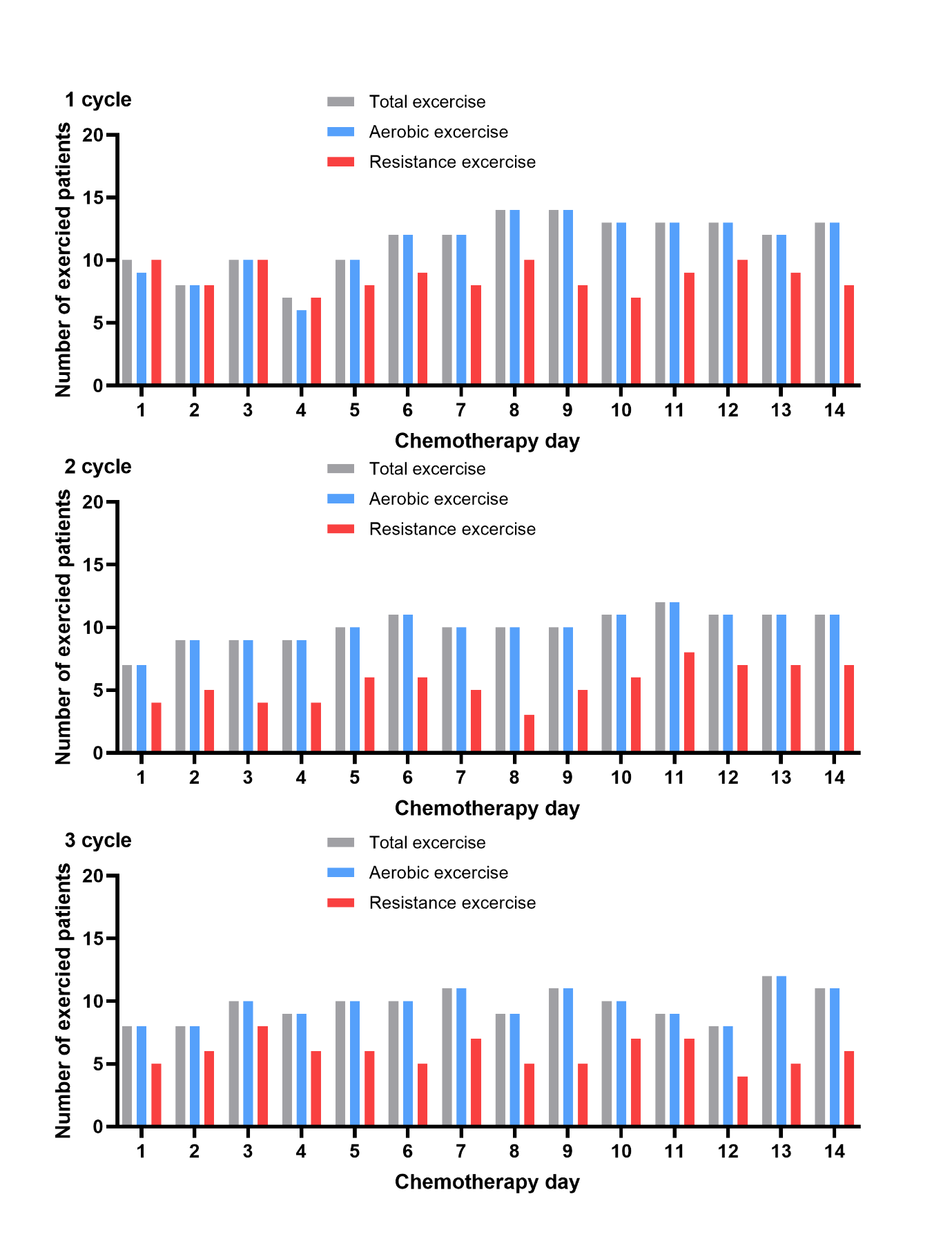


**Figure S4.** (a–c) Proportion of patients performing low-, moderate-, and high-intensity exercise during each chemotherapy cycle. (d–f) Location of exercise (hospital, home/near home, park, other) by chemotherapy day and cycle


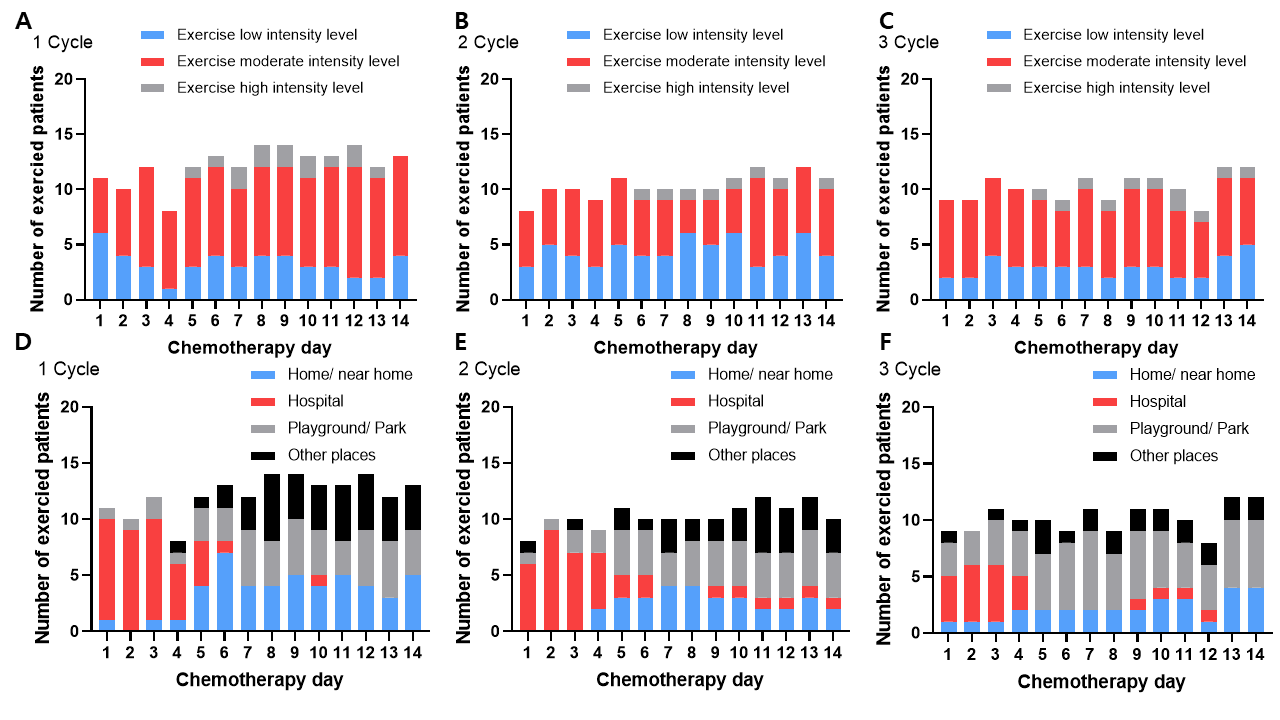

Supplement: Supplementary Table 1 — Changes in the European Organization for Research and Treatment of Cancer Quality of Life Questionnaire Core 30 (EORTC QLQ-C30) scores from baseline to week 12 during the exercise and nutrition intervention. [file Table1.docx]
